# Mobility Status Plays an Important Role in the Risk of Cardiovascular Rehospitalizations in Patients with Heart Failure Undergoing Cardiac Rehabilitation: A Retrospective Cohort Study

**DOI:** 10.3390/jpm12050675

**Published:** 2022-04-22

**Authors:** Po-Cheng Chen, Tsung-Hsun Yang, Po-Jui Wu, Lin-Yi Wang, Shyh-Ming Chen

**Affiliations:** 1 Department of Physical Medicine and Rehabilitation, Kaohsiung Chang Gung Memorial Hospital, College of Medicine, Chang Gung University, Kaohsiung City 833, Taiwan; b9302081@cgmh.org.tw (P.-C.C.); 8902077@cgmh.org.tw (T.-H.Y.); 2 Section of Cardiology, Department of Internal Medicine, Kaohsiung Chang Gung Memorial Hospital, College of Medicine, Chang Gung University, Kaohsiung City 833, Taiwan; sky1021@cgmh.org.tw; 3 Heart Failure Center, Kaohsiung Chang Gung Memorial Hospital, Kaohsiung City 833, Taiwan

**Keywords:** heart failure, cardiac rehabilitation, cardiovascular rehospitalization

## Abstract

The aim of this study was to investigate the association between mobility status and cardiovascular rehospitalizations in patients with heart failure undergoing cardiac rehabilitation. This retrospective cohort study included patients with heart failure undergoing cardiac rehabilitation. Mobility status was evaluated using functional ambulation categories (FAC), and each cardiovascular hospitalization was recorded by the case manager. A Poisson regression model was used to analyze the association between mobility status and cardiovascular rehospitalizations. This study included 154 patients with heart failure undergoing cardiac rehabilitation. For cardiovascular rehospitalizations within 6 months, the Poisson regression model reported that the impaired mobility group had a higher risk than the fair mobility group (incidence rate ratio (IRR) = 2.38, 95% CI 1.27–4.46, *p* = 0.007). For cardiovascular rehospitalizations within 12 months, the Poisson regression model also reported that the impaired mobility group had a higher risk than the fair mobility group (IRR = 1.91, 95% CI 1.16–3.13, *p* = 0.010). Other covariates, such as LVEF, peak oxygen consumption, and PAOD, could have impacted the risk of cardiovascular rehospitalizations. Among patients with heart failure undergoing cardiac rehabilitation, the impaired mobility group had a twofold risk of cardiovascular rehospitalizations, compared with the fair mobility group within both 6 and 12 months.

## 1. Introduction

Heart failure has gained increasing attention in Taiwan due to the high rate of hospitalizations and mortality. The rehospitalization rate of heart failure is 38.5%, while the all-cause mortality rate is 15.9% within one year after the index hospitalization in Taiwan according to the registry data of the Taiwan Society of Cardiology—Heart Failure with reduced Ejection Fraction (TSOC-HFrEF) [1]. Many patients are hospitalized for medical control of symptomatic heart failure, and some patients even require aggressive interventions, such as a left ventricular assistive device or heart transplantation. The above adverse events can be prevented through good care and education. In fact, heart failure disease management programs (HFDMPs) in Taiwan have improved the quality of care for patients with heart failure, and one study has reported fewer adverse events and treatment costs [2]. Disease management programs for heart failure consist of many factors, and cardiac rehabilitation plays an important role in controlling heart failure.

Multiple chronic conditions or morbidities adversely affect the cardiovascular outcomes in patients with heart failure. However, few studies have investigated the prognosis of impaired mobility in patients with heart failure. Physical conditions, such as arthritis or osteoporosis, increased the risk for death and hospitalizations in patients with heart failure, but the mobility status was not recorded in this study [3]. According to one population-based cohort study [4], impaired mobility, in addition to other noncardiac multimorbidity, could additively increase the risk of all-cause mortality in patients with HF. The above finding was not limited to elderly patients with heart failure and was also found in those aged less than 65 years old. Furthermore, the effect of impaired mobility on cardiovascular outcomes was also similar among both men and women and with varying heart functions [4]. Another study [5] also found that mobility disability was related to mortality in a geriatric population. Impaired mobility may prevent patients with heart failure from undergoing aggressive cardiac rehabilitation, which would also eventually deteriorate the heart failure prognosis.

We obtained no evidence regarding how much influence impaired mobility had on patients with heart failure undergoing cardiac rehabilitation. Our primary aim was to compare the risk of cardiovascular outcomes between fair and impaired mobility groups among patients with heart failure.

## 2. Materials and Methods

### 2.1. Study Design and Participants

This retrospective cohort study included patients with heart failure who underwent phase 2 cardiac rehabilitation from September 2010 to April 2018 at one tertiary medical center in southern Taiwan. These patients were diagnosed with acute decompensated heart failure by cardiologists and evaluated using the cardiopulmonary exercise test (CPET) once their clinical conditions became stable. After undergoing the CPET, patients were referred to the outpatient department (OPD) of physical medicine and rehabilitation (PMR) for phase 2 cardiac rehabilitation. All of the included patients underwent cardiac rehabilitation at least once and were followed up by a case manager for one year. Any cardiovascular rehospitalization was recorded during this one-year follow-up. This study was approved by the Institutional Review Board.

### 2.2. Cardiac Rehabilitation Programs

In this study, the training program for phase 2 cardiac rehabilitation followed the recommendations of the American College of Sports Medicine (ACSM) [6]. Moderate continuous training (MCT) of aerobic exercise was prescribed by a board-certified physiatrist specialized in cardiac rehabilitation. The types of exercise consisted of treadmill walk/walk–jog/jog, ergometer cycling, stair climber, and elliptical machine training. The exercise type was individually adjusted for patients with musculoskeletal or neurological disorders. The target intensity of training was 40–60% of peak oxygen consumption, or 10 beats below the heart rate associated with endpoints during the CPET, such as angina, drop in blood pressure, or significant ST depression. The training intensity increased gradually every two weeks to reach the target by a rate of perceived exertion (RPE) 12–14. The training duration was 40 min, with 5–10 min of warm-up and cool-down, respectively. The frequency of training was three sessions per week, and a complete course of exercise training was composed of 36 sessions. Telemetry electrocardiography (ECG) and oximeter monitoring were used in patients at higher risk, such as those with left ventricle ejection fraction (LVEF) < 30%, peak oxygen consumption < 14 mL/Kg/min, or significant ECG abnormalities during CPET. Resistance exercise involving both upper and lower extremities with the intensity of Borg rating of perceived exertion (RPE) 11–13, 10–15 repetitions per set, 1–3 sets per session, and 2–3 nonconsecutive days a week, was also prescribed after an uneventful MCT for 4 weeks. The physiatrist also prescribed flexibility exercises for patients pursuant to the recommendations of ACSM guidelines [6].

### 2.3. Exposure Variables

The mobility status of each patient was evaluated by the physiatrist at the first visit of OPD of PMR using functional ambulation categories (FAC) [7]. The FAC is a 6-point functional walking test that assesses how much human support patients require when walking, regardless of whether or not they use assistive devices. It ranges from a score of 0 to 5, in which 0: nonfunctional ambulatory; 1: requires continuous manual contact during ambulation; 2: requires intermittent or continuous light touch during ambulation; 3: ambulation on a level surface without manual contact with another person but requires standby guarding of one person; 4: independent ambulation on a level surface but requires supervision to negotiate; 5: independent ambulation everywhere. For analytical purposes, our investigators categorized these patients’ mobility status into two categories: fair mobility (FAC score 4–5) and impaired mobility (FAC score 0–3). Mobility status was usually associated with patients’ neurological and orthopedic comorbidities, so we also analyzed the distribution of these comorbidities.

### 2.4. Outcome Variables

The primary outcomes of this study were cardiovascular rehospitalizations within 6 and 12 months after cardiac rehabilitation. One case manager checked the records from the emergency department every week to determine whether any participant was hospitalized for cardiovascular events. Although death before rehospitalization is a competing risk for rehospitalization [8], we did not include this possible bias in the statistical analysis because death was a relatively infrequent event in our study.

### 2.5. Covariates

Demographic characteristics including age, sex, BMI, smoking habits (yes or no), and etiology and severity of heart failure were potential covariates in our study. The etiology of heart failure was categorized as ischemic versus nonischemic, and the severity of heart failure was classified according to the New York Heart Association (NYHA) Classification [9], with the categories defined as follows: class I, no limitation of physical activity; class II, slight limitation of physical activity, and ordinary physical activity results in fatigue, palpitation, or dyspnea; class III, has marked limitation of physical activity, and less than ordinary activity results in fatigue, palpitation, or dyspnea; class IV, unable to carry on any physical activity without discomfort, and symptoms of heart failure even at rest.

Covariates for further analysis in this study also included underlying diseases (such as hypertension, diabetes mellitus, dyslipidemia, chronic obstructive pulmonary disease (COPD), and chronic kidney disease), medical history (such as acute coronary syndrome (ACS), previous percutaneous coronary intervention (PCI), previous coronary artery bypass grafting (CABG), and peripheral arterial occlusive disease (PAOD)), examinations (such as results of echocardiography and cardiopulmonary exercise testing (CPET)), medication or treatment, and total rehabilitation sessions.

### 2.6. Statistical Analysis

To compare the baseline characteristics between the fair mobility and impaired mobility groups, we carried out the chi-squared test or Fisher’s exact test for categorical variables and the independent *t*-test or Mann–Whitney U test for continuous variables according to the normality test. Furthermore, the distribution of neurological and orthopedic comorbidities and total rehabilitation recessions between the fair and impaired mobility groups was compared using the chi-squared test or Fisher’s exact test. In order to realize the risk difference in cardiovascular diseases between the fair and impaired mobility groups, we used the chi-squared test or Fisher’s exact test to compare the number of patients and number of cardiovascular hospitalizations within 6 and 12 months after cardiac rehabilitation. Since patients with more cardiovascular rehospitalizations were considered to have a higher risk than those with fewer cardiovascular rehospitalizations, the number of cardiovascular rehospitalizations was treated as count data. A Poisson regression analysis was conducted to analyze the risk of cardiovascular rehospitalizations within 6 and 12 months after cardiac rehabilitation between the fair and impaired mobility groups. Statistical significance was set at a *p*-value less than 0.05, and statistical analyses were performed using SAS software version 9.4 (SAS Institute Inc., Cary, NC, USA).

## 3. Results

This study included 154 patients (including 122 patients with fair mobility and 32 patients with impaired mobility) with heart failure who underwent cardiac rehabilitation at least once and continued follow-up for one year. The BMI (25.68 ± 4.72 versus 23.68 ± 4.19, *p* = 0.031), the severity of heart failure (NYHA class) (*p* = 0.040), and history of PAOD (*p* = 0.010) differed significantly between the fair and impaired mobility groups. We found no significant difference in medication or treatment between the fair and impaired mobility groups (Table 1).

Neurological and orthopedic comorbidities were distributed differently between the fair and impaired mobility groups (Table 2). The impaired mobility group had a significantly higher percentage of the central nervous system (CNS) (*p* = 0.014), peripheral nervous system (PNS) (*p* < 0.001), and orthopedic (*p* < 0.001) comorbidities than the fair mobility group. Of the study’s patients, 73% of the fair mobility group had no neurological or orthopedic comorbidity, but 97% of the impaired mobility group had at least one neurological or orthopedic comorbidity. Despite the differences in the distribution of neurological and orthopedic comorbidities, the number of total rehabilitation sessions was similar between the fair and impaired mobility groups (*p* = 0.655).

Table 3 shows the number of patients and number of cardiovascular rehospitalizations within 6 and 12 months after cardiac rehabilitation. The percentage of patients readmitted to the hospital was significantly greater in the impaired mobility group both within 6 months (*p* = 0.001) and within 12 months (*p* = 0.029). The frequency of cardiovascular hospitalizations was also higher in the impaired mobility group within 6 months (*p* = 0.001). For cardiovascular rehospitalizations within 6 months after cardiac rehabilitation, the Poisson regression model reported that the impaired mobility group had a higher risk than the fair mobility group (incidence rate ratio (IRR) = 2.38, 95% CI 1.27–4.46, *p* = 0.007), and that other covariates may have impacted the risk (LVEF: IRR = 0.98, 95% CI 0.95–1.00, *p* = 0.029; peak oxygen consumption: IRR = 0.92, 95% CI 0.86–0.99, *p* = 0.024). For cardiovascular rehospitalizations within 12 months after cardiac rehabilitation, the Poisson regression model also reported that the impaired mobility group had a higher risk than the fair mobility group (incidence rate ratio (IRR) = 1.91, 95% CI 1.16–3.13, *p* = 0.010), and that other covariates may affect the risk (LVEF: IRR = 0.98, 95% CI 0.96–0.99, *p* = 0.004; peak oxygen consumption: IRR = 0.90, 95% CI 0.85–0.95, *p* < 0.001; PAOD: IRR = 1.98, 95% CI 1.04–3.78, *p* = 0.039)(Table 4).

## 4. Discussion

Our study showed that the percentages of CNS, PNS, and orthopedic comorbidities were higher in the impaired mobility group than in the fair mobility group. Besides the percentage of patients, the frequency of cardiovascular hospitalizations was also significantly higher in the impaired mobility group within 6 months after cardiac rehabilitation. The frequency of cardiovascular hospitalizations was also higher in the impaired mobility group within 12 months after cardiac rehabilitation, but the difference did not reach statistical significance. The impaired mobility group had at least a twofold higher incidence rate of cardiovascular rehospitalizations than the fair mobility group within both 6 and 12 months. Other covariates, such as LVEF, peak oxygen consumption, and PAOD, may have impacted the risk of cardiovascular rehospitalizations.

Mobility impairment is considered to be associated with multiple cardiovascular diseases [10,11]. In the SNAC-K study (a Swedish national study) [10], the risk of impaired mobility increased linearly with the increasing number of cardiovascular diseases (such as ischemic heart disease, atrial fibrillation, heart failure, and stroke), and a dose–response relationship between impaired mobility and number of cardiovascular diseases was observed. Individuals with two or more cardiovascular diseases had more than a threefold increased likelihood of impaired mobility than individuals without cardiovascular disease. In another longitudinal self-report survey of the Behavioral Risk Factor Surveillance Survey (BRFSS) in the US [11], statistically significant relationships between impaired mobility and concomitant cardiovascular diseases were identified. One likely explanation is that impaired mobility may impede these people from engaging in health-promoting activities or obtaining preventive counseling and services, or may delay modification of important risk factors through medical, nursing, and other healthcare-provider-driven interventions, thus increasing the risk of cardiovascular diseases.

Previous studies have investigated the adverse effect of noncardiac comorbidities on patients with heart failure [12,13]. According to one study using administrative claims data, impaired mobility was related to a slightly higher risk of death in patients hospitalized due to acute myocardial infarction during the first year postdischarge [14]. Those with multicomorbidity alone and those with impaired mobility alone shared a similar risk of hospitalization and emergency department visits among patients with heart failure [15]. However, those with combined multicomorbidity and impaired mobility had the highest risk of death and excess healthcare utilization. Furthermore, Tisminetzky et al. [4] found an additive effect of impaired mobility beyond the burden of noncardiac multimorbidity on hospitalization or mortality. In our opinion, impaired mobility can lead to structural changes in the cardiovascular system, including the reduction in venous return, increased heart rate, and deconditioning, which, in turn, affect cardiac function and increase the risk of cardiovascular events. In our study, we also investigated the relationship between cardiovascular rehospitalizations and cardiopulmonary parameters in addition to noncardiac comorbidity or mobility status. Our investigation was able to quantify the risk of cardiovascular rehospitalizations more clearly by measuring these cardiopulmonary parameters.

Recent publications also addressed the role of physical disability or frailty in patients with heart failure [16,17,18]. Uchmanowicz et al. [17] reported that deterioration of functional capabilities and an increase in symptom severity naturally lead to increased hospitalization frequency in patients with heart failure. In one multicenter, randomized, controlled trial [16], early, transitional, tailored, progressive cardiac rehabilitation programs resulted in greater improvement in physical function than usual care, but the difference in the risk of cardiovascular rehospitalizations or death rate was statistically insignificant between two groups. In the published study from 2021 [16], logistic regression was used to compare the risk of cardiac rehospitalizations or death rates between two groups. However, we used Poisson regression for statistical analysis in our study because the increased frequency of cardiovascular rehospitalizations per person was believed to be related to poorer cardiovascular prognosis according to our clinical practice experiences. This statistical analysis could make the effect size more prominent. Another study [18] reported that oral supplementation of L-arginine potentiates the response to cardiac rehabilitation after myocardial infarction and cardiac revascularization, which can improve the physical function of patients with heart failure. The possible rationale was that L-Arginine is the substrate used by nitric oxide (NO) synthase to generate NO, and it has been shown to exert its beneficial effects on endothelium driving vasodilatation, reducing inflammation, and ameliorating physical function.

Few studies have discussed the role of impaired mobility in patients with heart failure who undergo cardiac rehabilitation. Our study reported a twofold risk of cardiovascular rehospitalizations in patients with impaired mobility compared to patients with fair mobility after adjusting for other covariates. We also used Poisson regression for statistical analysis because the increased frequency of cardiovascular rehospitalizations was believed to be related to poorer cardiovascular prognosis according to our clinical practice experiences. Nevertheless, our study still had several limitations. First, the retrospective design and the potential for unmeasured confounders may have resulted in bias in our findings. For example, the quality of each session of cardiac rehabilitation, detailed smoking status (current/former/never), medical compliance, and health behavior may affect the cardiovascular outcomes of each patient with heart failure, but none of these factors were measured in our study. In fact, such factors are difficult to record and measure in most studies. Second, cardiovascular hospitalizations were only recorded by the case manager in our hospital, so cardiovascular rehospitalizations at other hospitals may have been omitted, and the risks would be underestimated. However, patients with heart failure would be sent to the same hospital in case of any suspected cardiovascular event in the health system in Taiwan, so any omitted records would likely be insignificant. Third, although the number of rehabilitation sessions showed no statistical difference between the impaired and fair groups, the variations in the number of rehabilitation sessions within each group still cannot be ignored. Strict control of the number of rehabilitation sessions should be required in future studies.

## 5. Conclusions

In this study, among patients with heart failure undergoing cardiac rehabilitation, the impaired mobility group had a twofold risk of cardiovascular rehospitalizations compared to the fair mobility group within both 6 and 12 months. Other covariates, such as LVEF, peak oxygen consumption, and PAOD, may have also impacted the risk of cardiovascular rehospitalizations.

## Figures and Tables

**Table 1:** Baseline characteristics of the patients.

	Fair Mobility (*n* = 122)	Impaired Mobility (*n* = 32)	*p*-Value
**Age**	57.36 ± 13.23	61.00 ± 9.14	0.075
**BMI**	25.68 ± 4.72	23.68 ± 4.19	0.031 *
**Sex (Male:Female)**	99:23	22:10	0.149
**Smoking (Yes:No)**	49:73	11:21	0.684
**Etiology (ischemic:nonischemic)**	76:46	21:11	0.838
**NYHA class**			
I	10	2	0.040 *
II	73	12	
III	39	18	
**Underlying diseases (Yes:No)**			
**Hypertension**	77:45	21:11	0.839
**Diabetes mellitus**	48:74	15:17	0.545
**Dyslipidemia**	73:49	19:13	>0.999
**COPD**	9:113	3:29	0.714
**Chronic kidney disease**	100:22	28:4	0.600
**Medical history (Yes:No)**			
**History of ACS (in the past 12 months)**	33:89	8:24	>0.999
**Previous PCI**	21:101	3:29	0.412
**Previous CABG**	7:115	5:27	0.129
**PAOD**	3:119	5:27	0.010 *
**Examinations**			
**Echocardiography**			
**LVEF**	38.19±16.33	43.66±17.10	0.098
**CPET**			
**Peak oxygen consumption (mL/Kg)**	15.59±4.53	14.00±3.92	0.072
**Medication/Treatment (Yes:No)**			
**Antiplatelet**	87:35	20:12	0.390
**Anticoagulant**	23:99	11:21	0.091
**ACEI/ARB**	107:15	25:7	0.168
**β-blocker**	101:21	24:8	0.318
**Potassium-sparing diuretics**	47:75	8:24	0.214
**Loop diuretics**	68:54	13:19	0.164
**Digoxin**	17:105	5:27	0.781
**Amiodarone**	14:108	6:26	0.373
**ICD**	3:119	2:30	0.278
**CRT**	3:119	0:32	>0.999

All values are reported as mean ± standard deviation for continuous variables or count numbers for categorical variables. * *p* < 0.05. Abbreviations: BMI, body mass index; NYHA, New York Heart Association; COPD, chronic obstructive pulmonary disease; ACS, acute coronary syndrome; PCI, percutaneous coronary intervention; CABG, coronary artery bypass grafting; PAOD, peripheral arterial occlusive disease; LVEF, left ventricular ejection fraction; CPET, cardiopulmonary exercise testing; ACEI/ARB, angiotensin converted enzyme inhibitor/angiotensin receptor blocker; ICD, implantable cardioverter–defibrillator; CRT, cardiac resynchronization therapy.

**Table 2:** Distribution of neurological and orthopedic comorbidities between fair and impaired mobility groups.

	**Fair Mobility (*n* = 122)**	**Impaired Mobility (*n* = 32)**	***p*-Value**
**CNS**			
Yes	20	12	0.014 *
No	102	20	
**PNS**			
Yes	5	11	<0.001 ***
No	117	21	
**Orthopedic**			
Yes	12	17	<0.001 ***
No	110	15	
	**Fair Mobility (N = 122)**	**Impaired Mobility (N = 32)**	***p*-Value**
**None of the above**	89	1	<0.001 ***
**Only one of the above**	29	22	
**Any two of the above**	4	9	
**All of the above**	0	0	
**Total rehabilitation sessions**	28.38 ± 20.89	30.19 ± 18.00	0.655

* *p* < 0.05, ** *p* < 0.01, *** *p* < 0.001. Abbreviations: CNS, central nervous system; PNS, peripheral nervous system.

**Table 3:** Number of patients and number of cardiovascular rehospitalizations within 6 and 12 months after cardiac rehabilitation.

**(a) Number of Patients**	**Fair Mobility (*n* = 122)**	**Impaired Mobility (*n* = 32)**	***p*-Value**
**Within 6 months**			
No	105	18	0.001 **
Yes	17	14	
**Within 12 months**			
No	91	17	0.029 *
Yes	31	15	
**(b) Number of Hospital Readmissions**	**Fair Mobility (N = 122)**	**Impaired Mobility (N = 32)**	***p*-Value**
**Within 6 months**			
0	105	18	0.001 **
1	12	10	
≥2	5	4	
**Within 12 months**			
0	91	17	0.061
1	19	9	
≥2	12	6	

* *p* < 0.05, ** *p* < 0.01.

**Table 4:** Poisson regression model for cardiovascular rehospitalizations within 6 and 12 months after cardiac rehabilitation.

**Within 6 Months**					
**Parameter**	**Estimate**	**SE**	**IRR**	**95% CI of IRR**	***p*-Value**
**Intercept**	0.57	0.64			0.368
**Mobility status**					
Impaired	0.87	0.32	2.38	(1.27, 4.46)	0.007 **
Fair	0.00	0.00	1.00		
**LVEF**	−0.02	0.01	0.98	(0.95, 1.00)	0.029 *
**Peak oxygen consumption (mL/Kg)**	−0.08	0.04	0.92	(0.86, 0.99)	0.024 *
**PAOD**					
Yes	0.59	0.43	1.80	(0.77, 4.17)	0.174
No	0.00	0.00	1.00		
**Within 12 Months**					
**Parameter**	**Estimate**	**SE**	**IRR**	**95% CI of IRR**	***p*-Value**
**Intercept**	2.10	0.80			0.008 **
**Mobility status**					
Impaired	0.65	0.25	1.91	(1.16, 3.13)	0.010 *
Fair	0.00	0.00	1.00		
**LVEF**	−0.02	0.01	0.98	(0.96, 0.99)	0.004 **
**Peak oxygen consumption (mL/Kg)**	−0.11	0.03	0.90	(0.85, 0.95)	<0.001 ***
**NYHA class**					
III	−0.71	0.53	0.49	(0.17, 1.40)	0.181
II	−0.40	0.49	0.67	(0.26, 1.77)	0.422
I	0.00	0.00	1.00		
**PAOD**					
Yes	0.68	0.33	1.98	1.04, 3.78)	0.039 *
No	0.00	0.00	1.00		

* *p* < 0.05, ** *p* < 0.01, *** *p* < 0.001. Abbreviations: NYHA, New York Heart Association; PAOD, peripheral arterial occlusive disease; LVEF, left ventricular ejection fraction; IRR, incidence rate ratio; SE, standard error; CI, confidence interval.

## Data Availability

The data that support the findings of this study are available from the corresponding author upon reasonable request.
